# Changes in natural killer cells and exhausted memory regulatory T Cells with corticosteroid therapy in acute autoimmune hepatitis

**DOI:** 10.1002/hep4.1163

**Published:** 2018-02-26

**Authors:** Hannah C. Jeffery, Manjit K. Braitch, Chris Bagnall, James Hodson, Louisa E. Jeffery, Rebecca E. Wawman, Lin Lee Wong, Jane Birtwistle, Helen Bartlett, Ansgar W. Lohse, Gideon M. Hirschfield, Jessica Dyson, David Jones, Stefan G. Hubscher, Paul Klenerman, David H. Adams, Ye H. Oo

**Affiliations:** ^1^ Centre for Liver Research, Institute of Immunology and Immunotherapy and National Institute of Health Research Inflammation Biomedical Research Centre Birmingham University of Birmingham Birmingham United Kingdom; ^2^ Human Biomaterials Resource Centre University of Birmingham United Kingdom; ^3^ Institute of Translational Medicine University Hospitals Birmingham National Health Services Foundation Trust, University of Birmingham Birmingham United Kingdom; ^4^ School of Life Sciences, Faculty of Health and Life Sciences Coventry University Coventry United Kingdom; ^5^ Newcastle Biomedical Research Centre and Newcastle University Newcastle United Kingdom; ^6^ Clinical Immunology Department University of Birmingham Birmingham United Kingdom; ^7^ University of Hamburg Hamburg Germany; ^8^ Liver Transplantation and Hepatobiliary Unit, Queen Elizabeth Hospital University Hospitals Birmingham National Health Service Foundation Trust Birmingham United Kingdom; ^9^ Department of Histopathology, Queen Elizabeth Hospital University Hospitals Birmingham National Health Service Foundation Trust Birmingham United Kingdom; ^10^ Peter Medawar Building of Pathogen Research University of Oxford Oxford United Kingdom

## Abstract

Autoimmune hepatitis (AIH) is an immune‐mediated liver disease currently treated by immunosuppressive medications with significant side effects. Thus, novel mechanistic treatments are greatly needed. We performed prospective deep immunophenotyping of blood immune cells in patients with acute AIH before and after corticosteroid therapy. Blood samples from 26 patients with acute AIH (United Kingdom‐AIH Consortium) were phenotyped by flow cytometry at baseline and 4 months after starting corticosteroids. Pretreatment liver tissues were stained for forkhead box P3‐positive (FOXP3^POS^) regulatory T cells (Tregs), clusters of differentiation (CD)56^POS^ natural killer (NK) cells, and chemokine (C‐X‐C motif) ligand 10. Chemokine secretion by cultured primary hepatocyte and biliary epithelial cells was measured by enzyme‐linked immunosorbent assay. Functional coculture assays with stimulated NK cells and Tregs were performed. CD161 ligand, lectin‐like transcript‐1 expression by intrahepatic immune cells was demonstrated with flow cytometry. Frequencies of NK^bright^ cells declined with therapy (*P* < 0.001) and correlated with levels of alanine aminotransferase (*P =* 0.023). The Treg:NK^bright^ ratio was lower pretreatment, and Tregs had an activated memory phenotype with high levels of CD39, cytotoxic T lymphocyte antigen 4, and FOXP3 but also high programmed death ligand 1, indicating exhaustion. Coculture experiments suggested the Tregs could not efficiently suppress interferon‐γ secretion by NK cells. Both Tregs and NK cells had high expression of liver infiltration and T helper 17 plasticity‐associated marker CD161 (*P =* 0.04). Pretreatment and CD161^pos^ NK cells expressed high levels of perforin and granzyme B, consistent with an activated effector phenotype (*P* < 0.05). Lectin‐like transcript 1, a ligand for CD161, is expressed on intrahepatic B cells, monocytes, and neutrophils. *Conclusion:* Activated effector NK cells, which correlate with biochemical measurements of hepatitis, and exhausted memory Tregs are increased in the blood of patients with treatment‐naive AIH and decline with corticosteroid therapy. Inadequate regulation of NK cells by exhausted FOXP3^pos^ Tregs may play a role in AIH pathogenesis and contribute to liver injury. (*Hepatology Communications* 2018;2:421‐436)

**Abbreviations**
AIH: autoimmune hepatitis
ALT: alanine aminotransferase
CCR7: chemokine (C‐C motif) receptor 7
CD: clusters of differentiation
CTLA‐4: cytotoxic T lymphocyte antigen 4
CXCL‐10: chemokine (C‐X‐C motif) ligand 10
CXCR3: cysteine‐X‐cysteine receptor 3
EM: effector memory
FOXP3: forkhead box P3
IFNγ: interferon‐γ
IgG: immunoglobulin G
IL: interleukin
LLT1: lectin‐like transcript 1
NK: natural killer
NKT: natural killer T cells
PD1: programmed death ligand 1
Th: T helper
TNFα: tumor necrosis factor α
Treg: regulatory T cell
UK‐AIH: United Kingdom Autoimmune Hepatitis
ULN: upper limit of normal

## Introduction

Autoimmune hepatitis (AIH) is an immune‐mediated liver disease characterized by interface and lobular hepatitis^1^ comprising infiltrates of both effector and regulatory T lymphocytes (Tregs).^1^, ^2^ There have been no new therapies for AIH for more than 3 decades, and it is becoming increasingly clear that there are limitations to the long‐term safety and efficacy of the nonspecific and empirical treatment in current use.^3^ Thus, there is a need for more effective mechanistically grounded approaches to treatment, and a better understanding of the immune make‐up of patients before they receive treatment is crucial for developing such novel immune cell/pathway‐targeted treatments for AIH.

One of the challenges in studying the immune status in patients who are treatment naive is the rapid initial response to corticosteroid treatment. This means that most patients are started on therapy before they can be investigated. In the vast majority of patients, this treatment is with corticosteroid or immunosuppressive therapy, which by nature alters immune activation status. Although studies have been performed to dissect the immune cell composition of patients with AIH on treatment, the immune balance between regulatory and effector cells in the treatment‐naive state and during longitudinal follow‐up of patients with acute AIH on maintenance immunosuppression is not known.

An imbalance between clusters of differentiation (CD)4^positive[pos]^CD25^pos^CD127^low^ Tregs^4^ and effector T cells has been proposed to contribute to the immune pathogenesis of AIH.^2^, ^5^, ^6^, ^7^ The differentiation and function of Tregs is controlled by transcription factor forkhead box P3 (FOXP3),^8^ and mutation in FOXP3 leads to a severe multiorgan autoimmune disorder (immunodysregulation polyendocrinopathy enteropathy X‐linked syndrome) in humans.^9^ CD56^pos^CD3^negative[neg]^ natural killer (NK) cells are a key component of the innate immune system and are involved in human autoimmune diseases, such as systemic lupus erythematosus^10^ and rheumatoid arthritis.^11^ NK cells are abundant in the liver.^12^ The activation and expansion of NK cells occurs in the early stages of AIH as NK cells function as a first response to liver injury^13^ and carry out diverse functions, including cytotoxicity, which may be directed at target cells, and cytokines interferon‐γ (IFNγ) and tumor necrosis factor α (TNFα) release, which can promote the maturation of antigen‐presenting cells to drive an adaptive immune response.^14^ Although NK cells have been shown to be particularly important in liver injury in viral hepatitis,^15^, ^16^ their frequency, function, and interaction with Tregs in the initial presentation of AIH, before corticosteroid therapy, and during longitudinal follow‐up is not known. In addition, recruitment and positioning of immune cells from peripheral circulation to the inflamed liver is also crucial for their function.^2^, ^17^

We hypothesized that dysregulation of effector innate NK cells and Tregs may play a role in acute AIH and that understanding this balance may help us to develop specific therapies and predict response to treatment. Based on this premise, we carried out a detailed, prospective, longitudinal immunophenotyping of immune cell subsets in patients with acute AIH before and 4 months after corticosteroid therapy and studied the influence of regulatory and effector lymphocytes on NK cells in functional assays. In addition, we screened for the presence of recruitment and positioning signals of liver‐infiltrated immune cells in treatment‐naive AIH livers.

## Materials and Methods

### ETHICS STATEMENT

Written informed consent was obtained from all subjects under United Kingdom‐AIH (UK‐AIH) ethics. Peripheral blood and liver tissues were collected with local research ethics committee approval (Newcastle, 14/LO/0303; Birmingham, CA/5192).

### BLOOD AND LIVER TISSUE

We used 10 mL ethylene diamine tetraacetic acid‐chelated peripheral blood samples from patients diagnosed with AIH. AIH was diagnosed according to international AIH scoring criteria,^18^ which included scores from serologic, virology, and histologic information. Samples were transported overnight to the National Institute for Health Research Biomedical Research Unit, University of Birmingham, for next day analysis of immune phenotype by flow cytometry.

Pretreatment, paraffin‐fixed, liver biopsy sections were obtained from 6 Birmingham patients for analysis by immunohistochemistry. Explanted diseased liver was obtained from patients undergoing liver transplantation for liver diseases, including primary sclerosing cholangitis, primary biliary cholangitis, alcoholic liver disease, and non alcoholic steatohepatitis; lymphocytes, hepatocytes, and biliary epithelial cells were isolated as described.^19^, ^20^, ^21^, ^22^, ^23^

### STUDY DESIGN

The UK‐AIH Consortium immunophenotyping study reported here was set up as a prospective assessment comparing the changes in a set of predefined immune cell subset features for consecutive treatment‐naive patients recruited between May 2015 and November 2016 and evaluating these features in relation to changes in a set of predefined biochemical factors that indicate liver function (alanine aminotransferase [ALT], bilirubin, and immunoglobulin G [IgG]). Immune features compared included the frequencies of nine predefined, innate, and adaptive immune cell subsets (CD4, CD8, CD4CD8, and CD4‐CD8‐double‐negative CD3+ T cells; CD127+CD25+ CD4 Tregs; CD56+ NK T cells (NKT); CD19+ B cells; CD56 bright NK [NK^bright^] and CD56 dim NK [NK^dim^] cells) and their expression of four surface markers (cysteine‐X‐cysteine receptor 3 [CXCR3], interleukin [IL]‐6R, CD161, programmed death ligand 1 [PD1]). The initial body of data indicated important changes in NK and Treg subsets; thus, additional analyses were initiated for subsequent patients to also compare the effector phenotypes of NK subsets and the frequencies and phenotypes of effector and memory populations of CD4, CD8 T cells, and Treg populations before and after steroid therapy.

### ISOLATION OF PERIPHERAL BLOOD MONONUCLEAR CELLS AND IMMUNE PHENOTYPING BY MULTICOLOR FLOW CYTOMETRY

Please see the http://onlinelibrary.wiley.com/doi/10.1002/hep4.1163/full.

### FLOW CYTOMETRY ANALYSIS OF LECTIN‐LIKE TRANSCRIPT‐1 EXPRESSION BY LIVER‐INFILTRATING IMMUNE CELLS

Please see the http://onlinelibrary.wiley.com/doi/10.1002/hep4.1163/full.

### IMMUNOHISTOCHEMISTRY STAINING OF CHEMOKINE (C‐X‐C MOTIF) LIGAND‐10, FOXP3, AND CD56 IN THE HUMAN LIVER

Pretreatment AIH liver biopsies were stained with anti‐chemokine (C‐X‐C motif) ligand 10 (CXCL‐10) (6D4; R&D Systems) and either anti‐FOXP3 (236A/E7; Abcam) or anti‐CD56 (CD564; Leica) on the Bond Rx system by using Bond Polymer Refine 3,3′‐diaminobenzidine tetrahydrochloride hydrate and Bond Polymer Refine red detection substrates (Novocastra).

### ENZYME‐LINKED IMMUNOSORBENT ASSAY OF CXCL‐10 SECRETION BY STIMULATED PRIMARY HUMAN HEPATOCYTES AND BILIARY EPITHELIAL CELLS

CXCL‐10 concentrations in culture supernatants were measured using a bead‐based Bio‐Plex Pro assay (Bio‐Rad) according to the manufacturer's instructions.

### NK AND T‐CELL COCULTURE ASSAYS

Please see the http://onlinelibrary.wiley.com/doi/10.1002/hep4.1163/full.

### CLINICAL DATA COLLECTION

ALT, bilirubin, and IgG data were collected for each patient before and 4 months after immunosuppressive therapy. All patients on the study received steroid therapy. There were differences in the specific formulation of steroid given depending on the local practice and whether the patient had cirrhosis (prednisolone) or not (budesonide). Steroid dosage followed national and international guidelines.

### STATISTICAL ANALYSIS

Comparisons between baseline and 4 months were performed using Wilcoxon tests. Changes between baseline and 4 months were compared to the changes in ALT, bilirubin, and IgG using Spearman's correlation coefficients. Comparisons across cell subsets or conditions of experiment were performed using Mann‐Whitney, Wilcoxon, or Friedman tests, with Dunn's post‐hoc analysis. Analyses were performed using SPSS 22 (IBM Corp., Armonk, NY) or GraphPad Prism software version 6 (San Diego, CA). Data were summarized graphically with error bars representing mean ± SEM. *P* < 0.05 was considered significant.

## Results

We studied the baseline and month 4 peripheral immunophenotype in 26 patients (3 male, 23 female; median age 56 years, range 44‐66 years) with serologically and histologically confirmed diagnosis of acute AIH from different National Health Service hospitals across the United Kingdom. These patients had clinical markers of disease activity with presenting ALT (369 IU/L, range 82‐1,352), bilirubin (26 μmol/L, range 10‐121), and IgG (23 mg/dL, range 14‐38). The detailed clinical characterizations of the patients reported in the study and the medications they received are given in http://onlinelibrary.wiley.com/doi/10.1002/hep4.1163/full.

### NK CELL FREQUENCIES ARE HIGHER IN PATIENTS WITH CORTICOSTEROID‐NAIVE AIH AND DECLINE WITH THERAPY

**Figure 1:**
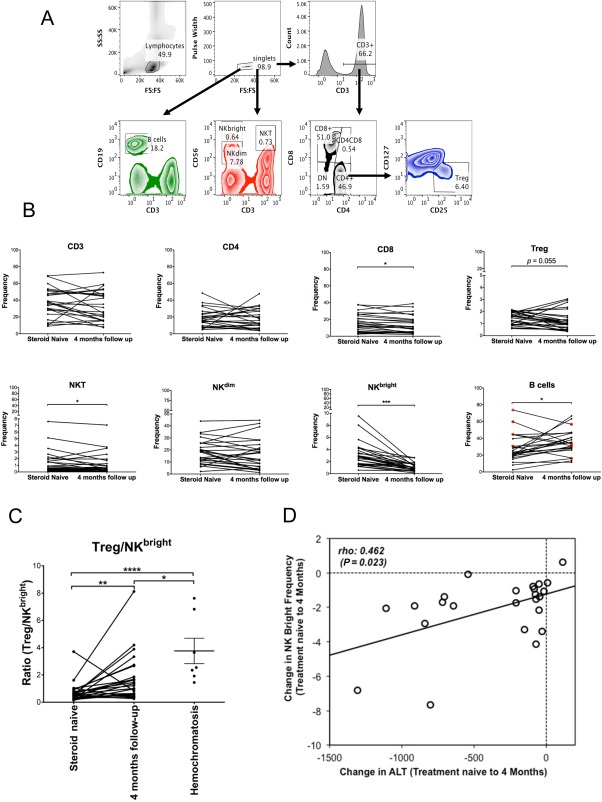
Peripheral blood immune cell frequencies in patients with AIH before and after 4 months immunosuppression therapy. (A) Gating strategy for definition of immune cell subsets, including CD4, CD8, CD4^‐^CD8^‐^ (double‐negative) T cells; CD4^pos^CD25^pos^CD127^neg^ regulatory T cells; CD56^pos^ T cells; NKT; CD19^+^ B cells; CD56^bright^ NK cells (NK^bright^), and CD56^dim^ NK cells (NK^dim^). (B) Cell subset frequencies as a proportion of total lymphocytes at baseline and after 4 months. Wilcoxon test, *P < 0.05, **P < 0.01, ***P < 0.0001. Red dots indicate patients with high starting B cell frequency that declined dramatically with therapy. (C) Ratio of Tregs to NK^bright^ cells in steroid‐naive, 4‐month follow‐up, and hemochromatosis (control) blood. Wilcoxon test, **P < 0.01; Mann‐Whitney U test, *P < 0.05, ****P < 0.0001. Error bars are means ± SEM. (D) Scatterplot and Spearman's correlation coefficient for the association between changes in ALT levels and NK^bright^ cell frequencies between baseline and 4 months. Trend line calculated by linear regression is shown.

To investigate changes in circulating immune cells in the 26 patients with acute AIH, we compared their peripheral blood immune cell profiles presented at diagnosis (treatment naive/baseline) and after 4 months on corticosteroid therapy. Subsets were defined by the gating strategy in Fig. 1A. Strikingly, frequencies of NK^bright^ cells were significantly higher in 25/26 patients at baseline and declined with therapy (median, 2.7% versus 0.9%; *P* < 0.001). Within the total lymphocyte pool, proportions of CD8 T cells (median, 14.4% versus 9.7%; *P =* 0.004; 20/26 patients), NKT (median, 0.63% versus 0.45%; *P =* 0.024; 19/26 patients), and Tregs (median, 1.6% versus 1.0%; *P =* 0.055; 19/26 patients) were also reduced with therapy (Fig. 1B). B‐cell frequencies were significantly lower at baseline (median, 23% versus 33%; *P =* 0.03), with increases after therapy observed in 80% of cases (20/25). Other cell subsets did not change significantly (Fig. 1A). Overall, the relative Treg/NK^bright^ balance increased significantly with therapy (Fig. 1C), and compared to control patients with hemochromatosis, the Treg/NK^bright^ ratio was significantly reduced in treatment‐naive patients (*P* < 0.0001). This was not only due to lower NK^bright^ frequencies in hemochromatosis but also due to higher Treg frequencies in controls compared to patients with AIH (http://onlinelibrary.wiley.com/doi/10.1002/hep4.1163/full). Furthermore, there was a significant correlation between the changes in ALT and frequencies of NK^bright^ cells from baseline to 4‐month follow‐up (*P =* 0.02) (Fig. 1D; http://onlinelibrary.wiley.com/doi/10.1002/hep4.1163/full). None of the other effector populations (CD8 and NKT) that declined with therapy altered significantly in their balance with Tregs (http://onlinelibrary.wiley.com/doi/10.1002/hep4.1163/full) or correlated with biochemical markers of disease activity (http://onlinelibrary.wiley.com/doi/10.1002/hep4.1163/full). Corticosteroid therapy was thus associated with a change in the immune balance toward a higher proportion of Tregs over NK^bright^ cells.

### BOTH REGULATORY MEMORY AND EFFECTOR MEMORY T‐CELL FREQUENCIES ARE SIGNIFICANTLY HIGHER IN THE CIRCULATION IN TREATMENT‐NAIVE PATIENTS WITH AIH AND DECLINE AFTER THERAPY

**Figure 2:**
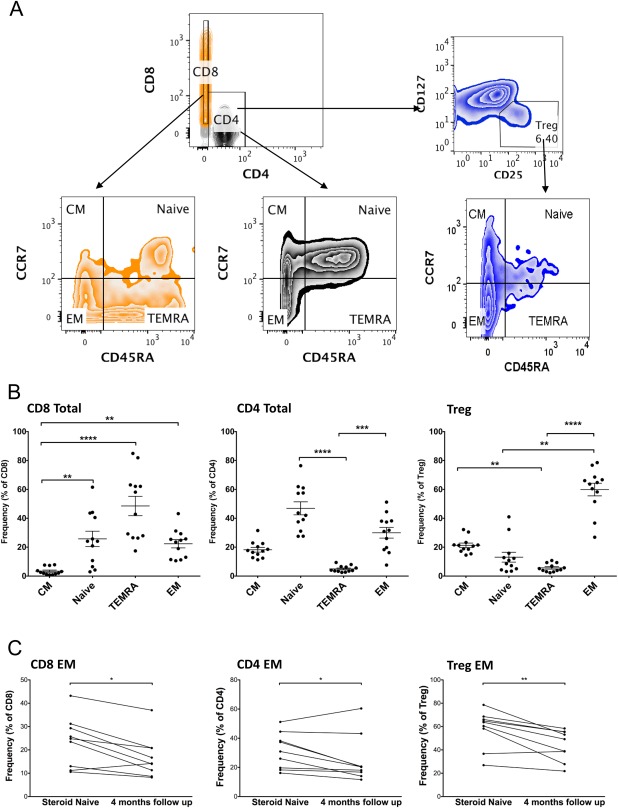
Frequencies of memory/naive CD8, CD4, and Treg subsets in treatment‐naive AIH before and after 4 months immunosuppression. (A) T‐cell memory and naive subsets were identified based on expression of CD45RA and CCR7. Representative fluorescence‐activated cell sorting plots illustrating the gating strategy are shown. (B) Frequencies before treatment. Friedman test with Dunn's post‐hoc analysis, *P < 0.05, **P < 0.01, ***P < 0.001, ****P < 0.0001. (C) Frequencies of EM CD4, CD8, and Tregs before and after 4 months immunosuppression. Wilcoxon test, *P < 0.05. Abbreviations: CM, central memory; TEMRA, terminally differentiated tissue resident effector memory RA‐positive.

We additionally explored whether T cells had been exposed to antigen by assessing the memory/naive status of the cells in a subgroup of the cohort (n = 12 treatment naive; n = 9 follow‐up). We defined memory and naive CD4, CD8, and Treg populations by their expression of CD45RA and chemokine (C‐C motif) receptor 7 (CCR7)^24^, ^25^ as central memory (CD45RA^neg^CCR7^pos^), effector memory (EM; CD45RA^neg^CCR7^neg^), naive (CD45RA^pos^CCR7^pos^), and tissue‐resident terminally differentiated effector memory RA^pos^ (TEMRA; CD45RA^pos^CCR7^neg^) (Fig. 2A). Within the CD4 and CD8 populations in the treatment‐naive state, naive CD4 T‐cell and TEMRA CD8 T‐cell subsets predominated (Fig. 2B). In contrast, the EM Treg subset was the significantly predominant Treg population (Fig. 2B). In all T‐cell classes, we observed significantly higher frequencies of EM cells at baseline compared to follow‐up (Fig. 2C).

### MEMORY Tregs EXPRESS SUPPRESSIVE FUNCTIONAL MARKERS BUT ALSO PRESENT AN EXHAUSTED PHENOTYPE IN PATIENTS WITH CORTICOSTEROID‐NAIVE AIH

**Figure 3:**
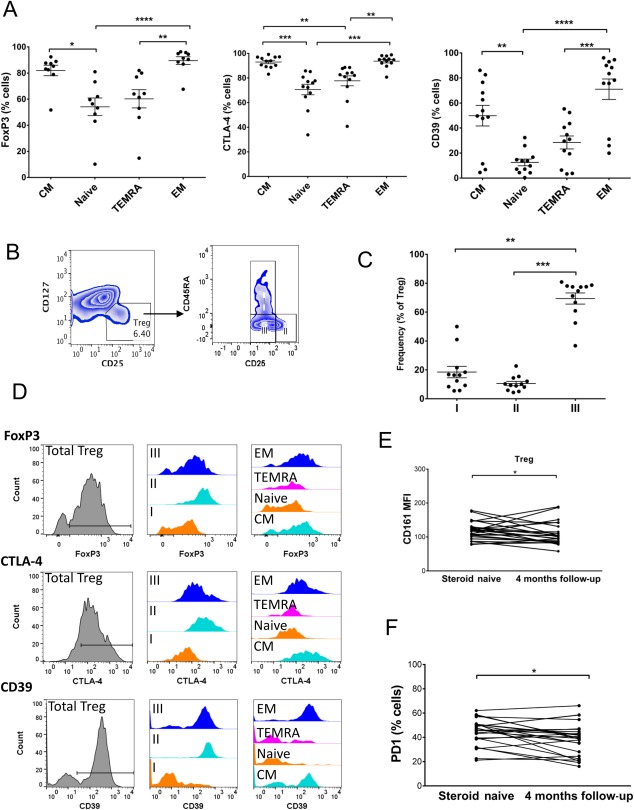
Treg phenotype in acute AIH. (A) Expression of Treg functional proteins FOXP3, CTLA‐4, and CD39 by memory/naive Treg subsets defined as in Fig. 2 by CD45RA and CCR7 expression. (B) Gating strategy for Treg fractions based on CD45RA and CD25 expression. (C) Frequencies of fractions in AIH pretreatment. In A and C, tests were Friedman with Dunn's post‐hoc analysis, *P < 0.05, **P < 0.01, ***P < 0.001, ****P < 0.0001. (D) Representative flow cytometry overlays showing expression of Treg functional proteins FOXP3, CTLA‐4, and CD39 by the total Treg population; fractions I, II, and III Tregs; and the memory and naive Treg subsets. (E) CD161 (MFI) and (F) PD1 (frequency) expression by total Tregs at baseline and after 4 months therapy. Wilcoxon test, *P < 0.05. Abbreviations: CM, central memory; MFI, median fluorescence intensity; TEMRA, terminally differentiated tissue resident effector memory RA‐positive.

FOXP3 transcription factor controls Treg function.^8^ Cytotoxic T lymphocyte antigen 4 (CTLA‐4) leads to transendocytosis of CD80/86 on dendritic cells to down‐regulate antigen presentation,^26^ and CD39 generates immunosuppressive adenosine.^27^ Memory Tregs had significantly higher expression of these markers compared to other Treg groups in the treatment‐naive patients (Fig. 3A,D; http://onlinelibrary.wiley.com/doi/10.1002/hep4.1163/full).

When we assessed the proportions of the Treg fractions defined by CD25 and CD45RA expression in treatment‐naive patients, we observed a predominance of fraction III (Fig. 3 B,C). However, fraction II had the highest level of Treg functional markers FOXP3, CTLA‐4, and CD39 (Fig. 3D; http://onlinelibrary.wiley.com/doi/10.1002/hep4.1163/full). Previous studies suggested that fraction III Tregs express CD161 and have a high propensity for differentiation toward a T helper (Th)17 phenotype.^25^, ^28^, ^29^ We found that CD161 expression on Tregs (median frequency, 12.9% versus 11.0%; *P =* 0.042; median fluorescence intensity, 118 versus 100; *P =* 0.037) was significantly higher before treatment (Fig. 3E). The frequency of CD161^pos^ Tregs was also higher before therapy compared to the 4‐month follow‐up (http://onlinelibrary.wiley.com/doi/10.1002/hep4.1163/full). Memory Tregs and fraction III Tregs had the highest expression of CD161 (http://onlinelibrary.wiley.com/doi/10.1002/hep4.1163/full). In addition, PD1, a marker of lymphocyte exhaustion, was reduced on Tregs after 4 months of immunosuppressive therapy (median, 46 versus 40; *P =* 0.027) (Fig. 3F).

### NK CELL CD161 EXPRESSION LEVELS ARE SIGNIFICANTLY HIGHER IN THE TREATMENT‐NAIVE STATE, AND CD161^pos^ NK CELLS ARE CHARACTERIZED BY HIGH EXPRESSION OF GRANZYME B AND PERFORIN

**Figure 4:**
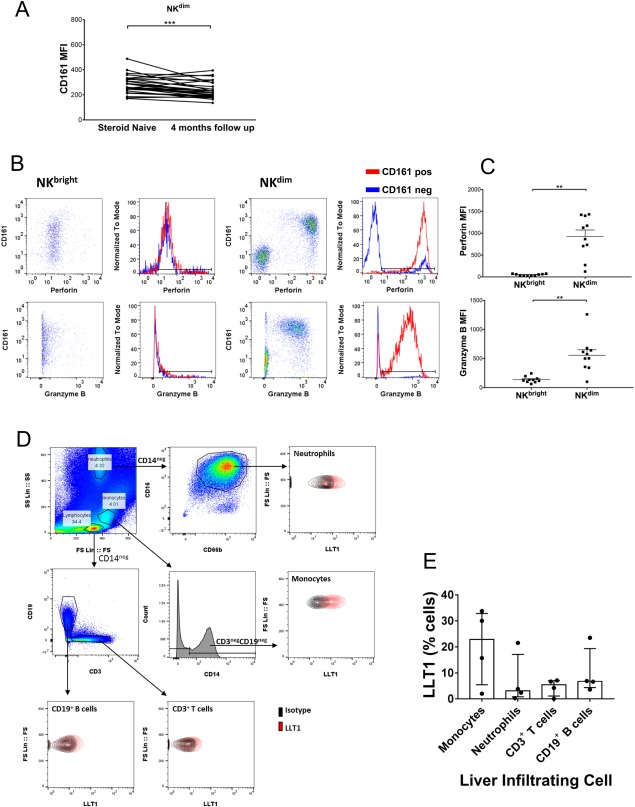
Characterization of CD161‐expressing NK cells and CD161 ligand LLT1 in AIH. (A) CD161 expression on NK^dim^ cells at baseline and after 4 months therapy. Wilcoxon test, *P < 0.05. (B) Representative flow cytometry plots and overlays showing granzyme B and perforin expression by CD161^pos^ and CD161^neg^ NK cell populations pretreatment. (C) Summary data on granzyme B and perforin expression by CD161^pos^ populations of NK^bright^ and NK^dim^ cells pretreatment; **P < 0.01. (D) Flow cytometry gating strategy for identification of immune cell subsets, including neutrophils, monocytes, T cells, and B cells, among isolated liver‐infiltrating immune cells and representative staining of LLT1 versus isotype control on these populations. (E) Summary data on LLT1 expression by liver‐infiltrating immune cells isolated from primary biliary cirrhosis, primary sclerosing cholangitis, alcoholic liver disease, and nonalcoholic steatohepatitis explanted livers. Abbreviation: MFI, median fluorescence intensity.

With the exception of CD19^pos^ B cells, all immune subsets contained CD161^pos^ cells (http://onlinelibrary.wiley.com/doi/10.1002/hep4.1163/full). The level of CD161 expression on NK^dim^ cells (median fluorescence intensity, 274 versus 217; *P* < 0.001) was significantly higher before treatment compared with after 4 months corticosteroid (Fig. 4A; http://onlinelibrary.wiley.com/doi/10.1002/hep4.1163/full). We characterized the expression of functional cytolytic molecules by NK subsets subdivided according to CD161 expression and observed the highest frequencies of cytotoxic factors granzyme B and perforin in the CD161^pos^ NK populations. CD161^pos^ NK^dim^ cells had significantly greater expression of both perforin and granzyme B on a cell by cell basis than NK^bright^ cells (Fig. 4B,C; http://onlinelibrary.wiley.com/doi/10.1002/hep4.1163/full).

Because we detected high frequencies of CD161^pos^ cells, we explored the expression of the CD161 ligand lectin‐like transcript 1 (LLT1), by liver‐infiltrating immune cells. We analyzed its expression by cells isolated from explanted autoimmune or inflammatory diseased liver tissue by flow cytometry (Fig. 4D) and detected LLT1 on T and B cells, monocytes, and neutrophils (Fig. 4E).

### Tregs AND NK CELLS IN TREATMENT‐NAIVE AIH SHOW HIGH EXPRESSION OF CXCR3, AND CXCR3 LIGAND CXCL‐10 DERIVES FROM INFLAMED LIVER HEPATOCYTES AND BILIARY CELLS

**Figure 5:**
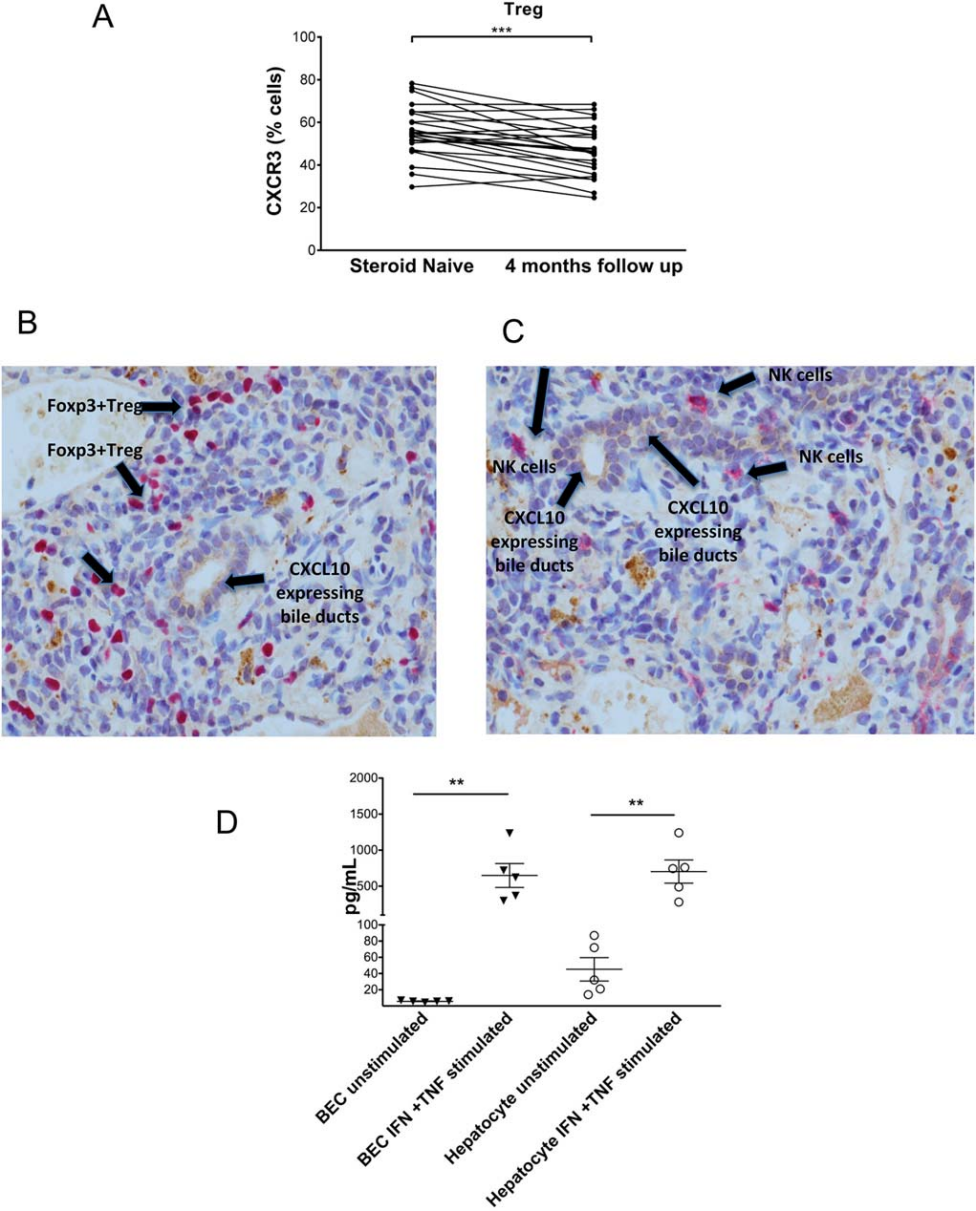
CXCL‐10 distribution in treatment‐naive AIH livers colocalizes with CD56+ NK cells and FOXP3+ Tregs. (A) CXCR3 expression by total Tregs at baseline and after 4 months therapy. Wilcoxon test, *P < 0.05. (B,C) Dual staining of CXCL‐10 and either (B) CD56 or (C) FOXP3 on liver biopsy sections taken at diagnosis of AIH (CXCL‐10, brown staining; FOXP3^+^Tregs, CD56^+^NK cells, red staining). (D) CXCL‐10 concentrations in culture supernatants prepared from untreated and IFNγ + TNFα‐treated hepatocytes and biliary epithelial cells. Data are mean ± SEM. Abbreviation: BEC, biliary epithelial cell.

CXCR3 was expressed by all subsets of immune cells, but expression levels varied (http://onlinelibrary.wiley.com/doi/10.1002/hep4.1163/full). CXCR3^pos^ Treg frequencies in the total Treg population were significantly higher in the treatment‐naive state than at follow‐up (*P* < 0.001) (Fig. 5A). The expression of CXCR3 was highest in the memory compared to naive Treg populations (*P* < 0.05) (http://onlinelibrary.wiley.com/doi/10.1002/hep4.1163/full). We have previously reported a role for CXCR3 in lymphocyte homing to the liver,^2^, ^17^, ^30^ but to confirm its potential involvement in the pathogenesis of acute AIH, we stained pretreatment liver biopsies for the CXCR3‐binding chemokine CXCL‐10. CXCL‐10 was detected on bile ducts in the portal tracts and was closely associated with infiltrating FOXP3^pos^ Treg cells (Fig. 5B) and CD56^pos^ NK cells (Fig. 5C). Furthermore, we demonstrated that CXCL‐10 was secreted by human hepatocytes and biliary epithelial cells in response to inflammatory cytokines *in vitro* (Fig. 5D).

### REGULATORY T‐CELL CONTROL OF NK CELLS

**Figure 6:**
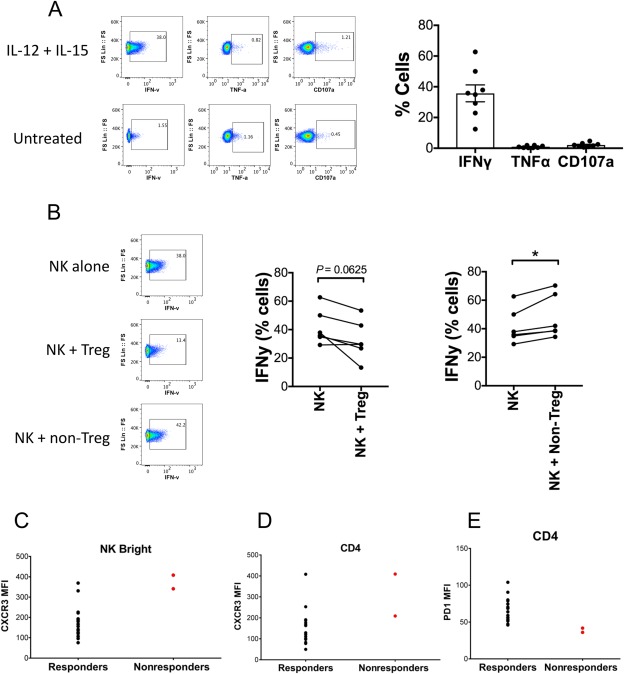
Regulation of NK cell function by activated regulatory and nonregulatory T cells. (A,B) NK cells, Tregs, and non‐Tregs were isolated from peripheral blood. NK cell degranulation (CD107a), IFNγ, and TNFα production, with overnight IL‐12 (20 ng/mL) + IL‐15 (20 ng/mL) stimulation monitored by flow cytometry in the presence or absence of activated Tregs or non‐Tregs. (A) Representative and summary data on NK degranulation and cytokine production with stimulation. (B) IFNγ production by NK cells cultured overnight alone or 1:1 with either Tregs or non‐Tregs under IL‐12 (20 ng/mL) + IL‐15 (20 ng/mL) stimulation. (C‐E) Expression of immune cell surface markers in responders and nonresponders after 4 months corticosteroid treatment. CXCR3 MFI on (C) NK^bright^ cells and (D) CD4 T cells. (E) PD1 MFI on CD4 T cells. Abbreviation: MFI, median fluorescence intensity.

To investigate whether the significant increase in the ratio of NK cells to Tregs could have functional implications in AIH pathogenesis, we tested whether activated NK cell effector responses could be controlled by Tregs *in vitro*. CD3^neg^CD56^pos^ NK cells activated overnight with cytokines IL‐12 and IL‐15 secreted high levels of IFNγ but expressed little TNFα or CD107a (Fig. 6A). In agreement with the idea that there is inadequate Treg control over NK in the AIH disease process, we observed a very clear trend of suppression of NK activity by Tregs (*P =* 0.0625); marked suppression by Tregs occurred in five out of the six experiments performed. This was in contrast to the effects of coculture of NK cells with activated non‐Treg effectors, which led instead to significantly elevated IFNγ expression by NK (*P =* 0.0312) (Fig. 6B).

### IMMUNOLOGICAL MARKERS AND PERIPHERAL BLOOD LIVER BIOCHEMISTRY AND IgG LEVEL

Finally, we explored whether any phenotypes examined in this study could define patients with treatment‐naive AIH who are nonresponders to corticosteroid therapy. We defined subjects as nonresponders based on their having 1.5 × upper limit of normal (ULN) values for ALT, 1.5 × ULN values for bilirubin, and 1.5 × ULN values for IgG at 4 months. Only 2 patients fulfilled these criteria, so any interpretation is limited; however, these patients were characterized by high levels of CXCR3 on their NK^bright^ cells and CD4 T cells (Fig. 6C,D) and lower expression of the exhaustion marker PD1 on CD4 T cells (Fig. 6E).

## Discussion

Type‐1 AIH is an immune‐mediated liver disease that usually responds to immunosuppression. Because patients are promptly treated with corticosteroids, it is difficult to study patients in the treatment‐naive state before immunosuppression is started. Our study is one of the first to characterize in detail the changes in immune cell composition before and after treatment in patients with AIH. The study was not designed to report the alterations in total cell numbers with therapy but rather to discuss the influence of therapy on the ratios of the different innate and adaptive immune subsets and to describe the changes in their individual phenotypes; as such, this study offers important insight into the subsets whose relative precedence may be key to AIH pathogenesis and thus crucial to target in future targeted therapies.

The two main findings of this study are a significant elevation of activated effector CD56^bright^ NK cells in the blood prior to therapy and a concomitant increase in regulatory T cells. In the healthy state, NK cells constitute 5%‐15% of peripheral blood cells and are abundant in the liver.^31^ NK cells have been implicated in liver injury^32^ and in the pathogenesis of autoimmune diseases, such as systemic lupus erythematosus and diabetes.^33^, ^34^ They provide an early host defense to infection or injury and have the ability to kill other cells rapidly by cytolysis through their release of cytolytic mediators without prior stimulation.^35^ We observed a significant increase in CD56^bright^ NK cells in untreated patients with AIH (25/26 cases) that fell after 4 months of immunosuppressive treatment. CD56^bright^ cells are found in the liver but under normal circumstances are present at low frequencies in the blood. They are critical for local innate immune responses and are increased at sites of inflammation in autoimmune diseases.^36^ We also observed a higher frequency of NK^dim^ cells (up to 40%) in treatment‐naive patients. It is possible that in treatment‐naive AIH, NK cells enter the peripheral circulation from the liver, but perhaps more likely, they are released into the blood from lymphoid tissues where they are found at high frequencies. In parallel to an increase in NK cell frequency, we also observed an increased Treg frequency in the circulation of patients with treatment‐naive AIH. Tregs are crucial to maintain peripheral immune tolerance and control effector immune cells.^37^, ^38^ The increase in the Treg population may thus be an attempt to suppress immune activation stimulated by the causative autoantigen in acute AIH. Although both NK^bright^ and Treg cell frequencies were increased in treatment‐naive patients, the Treg versus NK^bright^ cell ratio was reduced, suggesting a skewing of the immune balance toward the effector arm in the treatment‐naive state. Importantly, Tregs were weak suppressors of NK cell production of inflammatory cytokines; thus, the data suggest that loss of NK cell regulation could underlie hepatic damage in AIH. In support of this conclusion, the NK^bright^ cell frequency correlated with levels of ALT in treatment‐naive patients.

A previous *in vivo* study^39^ in diabetes suggested that Tregs suppress NK function by depleting IL‐2, which is critical for NK cell survival and optimal function, from the local environment. Although in the present study the suppression of NK cell production of inflammatory cytokines did not reach significance, there was a clear indication that Tregs offer a level of regulation over activated NK cells in the normal state. It is possible that in the treatment‐naive state of AIH, Tregs may be too exhausted, a conclusion supported by their elevated PD1 expression pretreatment, or too infrequent to effectively control NK cell activity. Overall, the data suggest that loss of NK cell regulation involving inadequate Treg‐mediated control could underlie hepatic damage in AIH. In support of this conclusion, the NK^bright^ cell frequency correlated with levels of ALT in treatment‐naive patients.

The changes in peripheral blood described in this study are informative, although it is likely that the most important responses pathologically are those that occur within the liver. Our data suggest that the expanded peripheral populations we have seen have the potential to be recruited to the inflamed liver because they express high levels of the chemokine receptor CXCR3, which facilitates the recruitment of effector and regulatory T cells to the inflamed human liver^2^, ^30^ through interactions with its ligand CXCL‐10.^2^, ^17^, ^30^ We detected both CXCR3^pos^ Tregs and NK cells residing close to CXCL‐10‐expressing bile ducts in liver biopsies from patients with acute AIH.

We examined the Treg subset in treatment‐naive patients in more detail and found that these cells were predominantly CD45RA^neg^ memory, which suggests that they have been exposed to antigen. Specifically, Tregs and also CD4 and CD8 cells were predominantly of an EM phenotype before exposure to immunosuppression, and this proportion declined significantly in all subsets after therapy. Thus, it is likely that the EM component of Tregs is reflective of extensive efforts to suppress antigen‐primed effector T cells in the liver.^40^ Future studies into the T‐cell receptor repertoire of both CD4 and Tregs in treatment‐naive AIH would be of interest to determine whether these cells are clonally expanded subsets that respond to the same antigen. In addition, Treg functional markers FOXP3, CD39, and CTLA‐4 were highest on the memory population, suggesting that these are suppressive memory Tregs. However, these cells also expressed high levels of PD1, which has been associated with T‐cell exhaustion, suggesting that these cells may not be fully functional. This could explain in part why immune‐mediated liver injury progresses in spite of increased Treg frequencies.

Treg cells in peripheral blood can be divided into three fractions,^25^ with fraction III Tregs characterized by the expression of CD161,^28^, ^29^ which has been described as a potentially plastic Treg fraction that can change its lineage toward Th17.^28^, ^29^ CD161 is a lectin‐like receptor expressed on human NK cells and T lymphocytes.^41^ We have previously demonstrated that CD161^pos^ T cells in the human liver include both Th17 and Tc17 cells^42^, ^43^ and that these cells are implicated not only in liver injury but also in the pathogenesis of autoimmune Crohn's disease.^44^ LLT1, a ligand for CD161,^45^ is present on antigen‐presenting cells, germinal center B cells, and Kupffer cells.^46^, ^47^ Interactions between CD161 on NK cells and LLT1 on target cells inhibit NK cell‐mediated cytotoxicity and cytokine production.^46^, ^47^, ^48^, ^49^ We observed significantly higher CD161 expression on both NK cells and Tregs in treatment‐naive AIH that declined after therapy and demonstrated that LLT1 is expressed on liver‐infiltrating monocytes, B cells, and neutrophils. Thus, CD161 could be activated by ligand‐bearing cells in the inflamed liver to down‐regulate effector responses; but if insufficient ligand is present, this feedback could be overwhelmed.

We noted that patients who did not respond to immunosuppressive therapy had a reduced exhausted T‐cell phenotype with lower PD1 expression, which agrees with a lack of control over effector cells in these patients with therapy. The B cell frequency was elevated in 20% (5/25 cases) of treatment‐naive patients, and this group might be one that would respond to anti‐B cell therapy, such as rituximab.^39^, ^50^

Our study has its limitations. As only small volumes of blood (5‐10 mL) were supplied by each participating hospital, the full volume was taken to isolate the peripheral blood mononuclear cells for immune phenotyping; count data on each subset in the whole blood were not collected. CD45 selection and dead cell exclusion were not included in the phenotyping panel. This decision was taken in the study design in order to maximize the depth of immune subset phenotyping with the small volume of sample available and access to only nine color instruments; it was also based on knowledge that CD45 frequencies of >99% and dead cell frequencies of 1%‐3% are consistently observed with peripheral blood samples processed under the same conditions and are seen to have negligible impact on the frequencies of the populations of interest in the study (http://onlinelibrary.wiley.com/doi/10.1002/hep4.1163/full; http://onlinelibrary.wiley.com/doi/10.1002/hep4.1163/full). There were differences in the steroid regimen used by different contributing centers, and these might have influenced the magnitude of phenotypic change observed. However, an increase in variability would be more reasonable to expect; as such, the conclusions of disease‐relevant phenotypes made in this study are believed to be sound and relevant. Limitation of sample volume to under 10 mL with the current UK‐AIH ethics meant that *in vitro* assays to test the possibility that a lack of NK cell regulation by Tregs contributes to AIH pathogenesis could not be evaluated on patient blood as such analysis required around 50 mL. Despite these limitations, the study has identified interesting and novel phenotypic changes with therapy in a unique, rare, and poorly understood cohort, which can now guide the direction of further studies to address in more detail the pathologic mechanisms underlying AIH pathogenesis and direct improved and stratified therapeutic strategies with reduced side effects.

**Figure 7:**
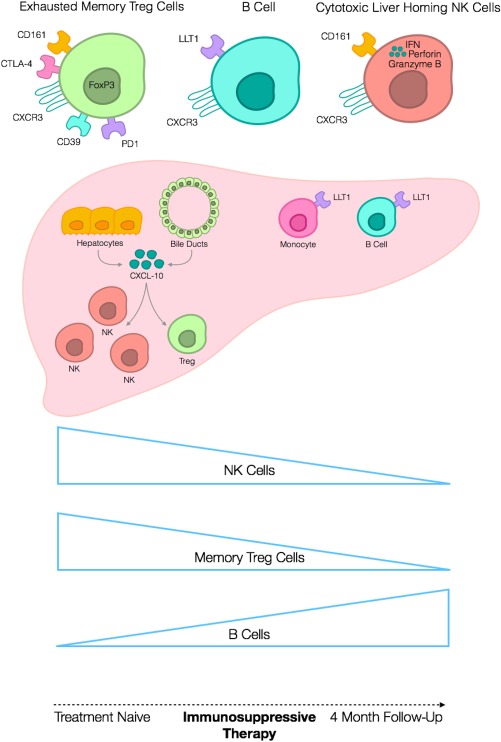
Diagrammatic illustration of the immune cell composition changes occurring in treatment‐naive AIH with 4 months corticosteroid therapy. Regulatory T cells in the peripheral blood of patients with treatment‐naive acute AIH are predominantly effector memory in phenotype (CD45RA^neg^CCR7^neg^) and express functional proteins CD39, CTLA‐4, and FOXP3. They also express the liver‐homing receptor CXCR3 and exhaustion marker PD1. B cells and monocytes in inflamed AIH livers express the CD161 ligand LLT1, and CD161 expression by both NK cells and Tregs is elevated before therapy in patients with acute AIH. CXCL‐10 secreted by inflamed hepatocytes and bile ducts attracts CXCR3‐expressing NK cells and Tregs to the site of hepatitis in AIH. Both NK cells and memory Tregs are expanded significantly in the peripheral immune cell composite of patients with acute AIH before the start of therapy compared to after 4 months on corticosteroid. In contrast, the B cell frequency is significantly lower at the treatment‐naive acute stage and recovers with corticosteroid.

In conclusion, our study is the first to report longitudinal changes in detailed leucocyte subsets in AIH (Fig. 7). The changes in the balance of NK cells and Tregs in treatment‐naive patients suggest a switch in the effector/regulatory balance that might be amenable to therapy. The relevance of the NK cell changes is supported by the correlation with liver injury early in disease. Further studies using deep immunophenotyping with mass cytometry in well‐characterized cohorts of patients are required before immunophenotyping can be used for treatment stratification.

Author names in bold designate shared co‐first authorship.

## Supporting information

Additional Supporting Information may be found at http://onlinelibrary.wiley.com/doi/10.1002/hep4.1163/full.

Supporting Information Figure 1

Supporting Information Figure 2

Supporting Information Figure 3

Supporting Information Figure 4

Supporting Information Figure 5

Supporting Information Figure 6

Supporting Information Figure 7

Supporting Information Figure 8

Supporting Information Figure 9

Supporting Information Figure 10

Supporting Information Figures

Supporting Information Tables

Supporting Information
