# Supplementary figures and images for: Changes in natural killer cells and exhausted memory regulatory T Cells with corticosteroid therapy in acute autoimmune hepatitis
Source: Hepatol Commun. 2018 Feb 26;2(4):421–36. doi: 10.1002/hep4.1163 (PMC5880196; doi:10.1002/hep4.1163)

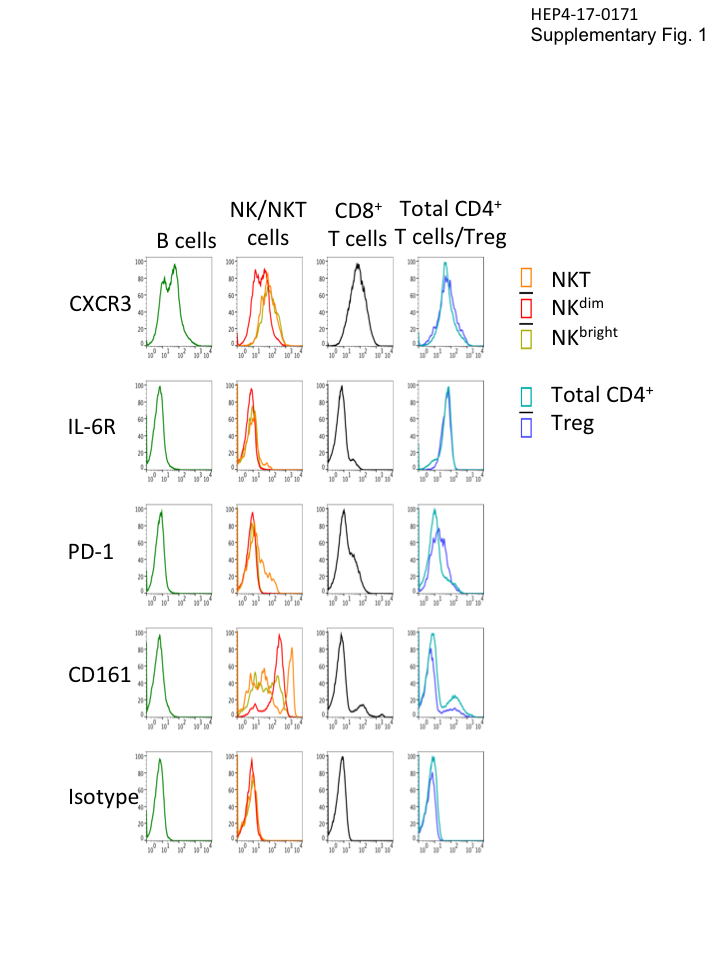

Supplement: Supplementary file 1 — Supporting Information Figure 1 [file HEP4-2-421-s001.tif]

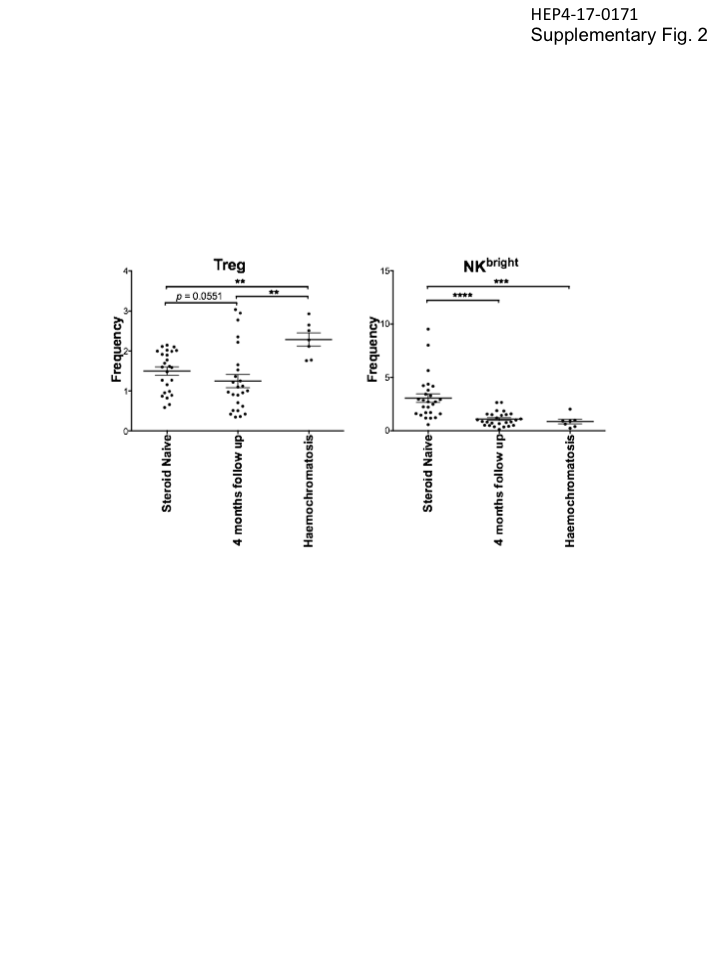

Supplement: Supplementary file 2 — Supporting Information Figure 2 [file HEP4-2-421-s002.tif]

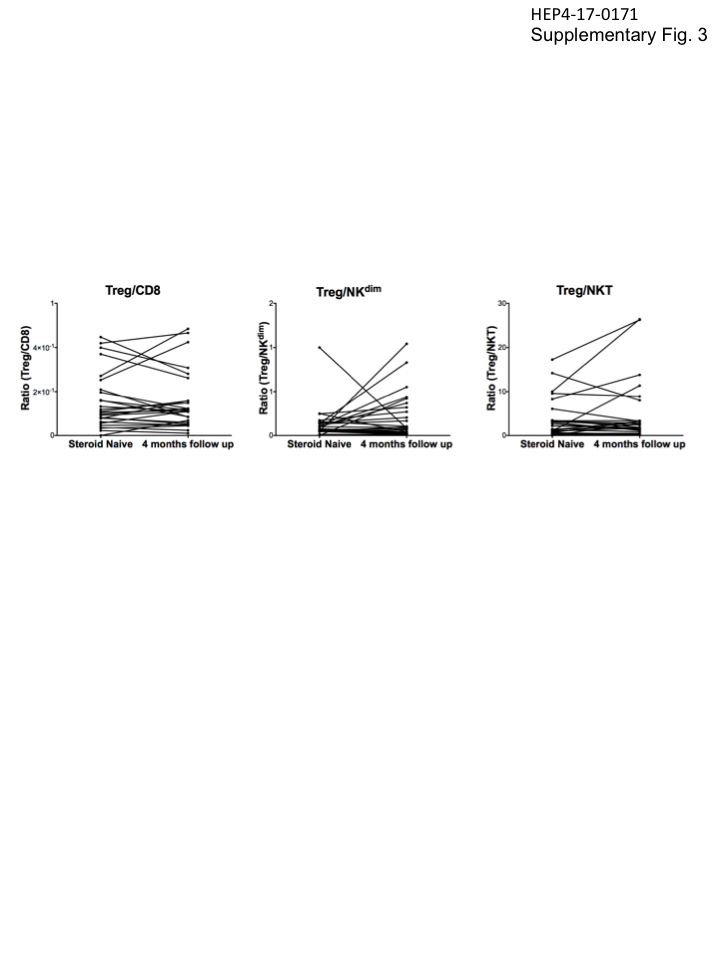

Supplement: Supplementary file 3 — Supporting Information Figure 3 [file HEP4-2-421-s003.tif]

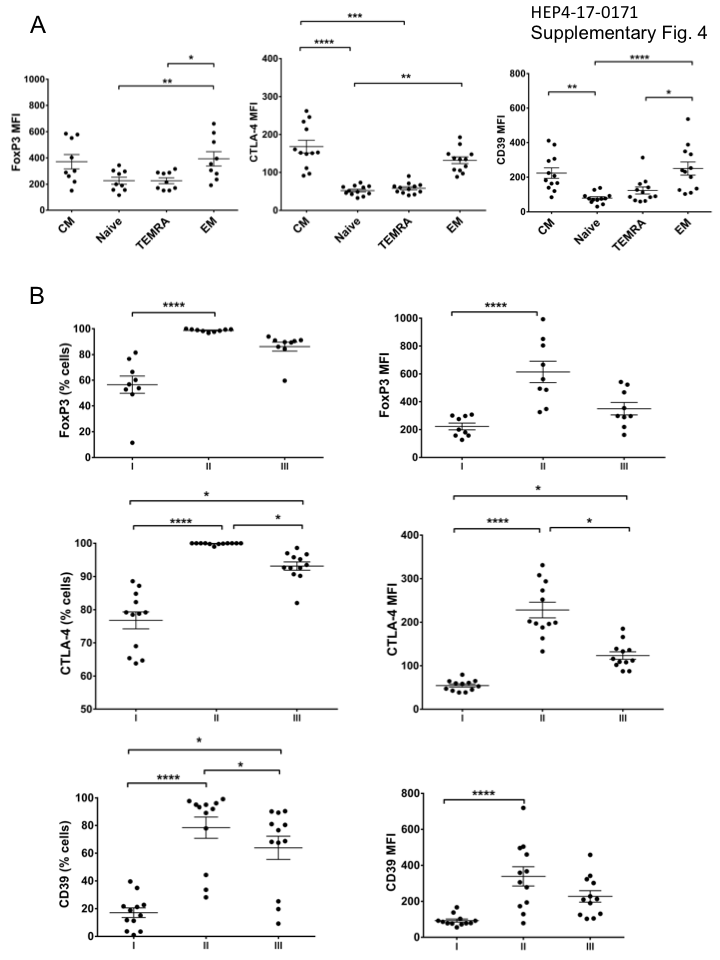

Supplement: Supplementary file 4 — Supporting Information Figure 4 [file HEP4-2-421-s004.tif]

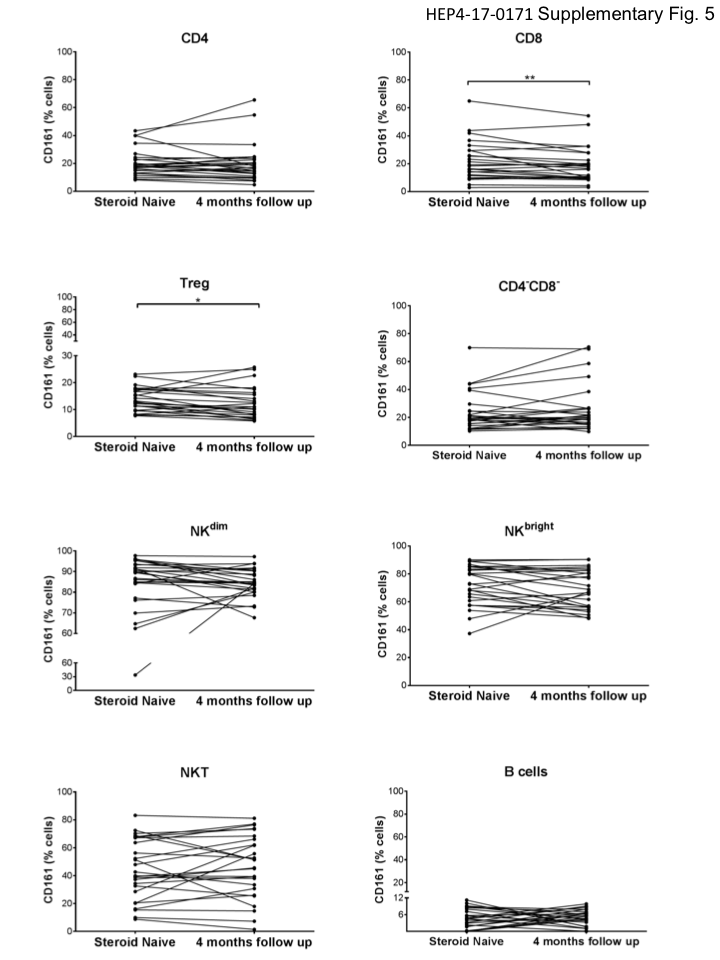

Supplement: Supplementary file 5 — Supporting Information Figure 5 [file HEP4-2-421-s005.tif]

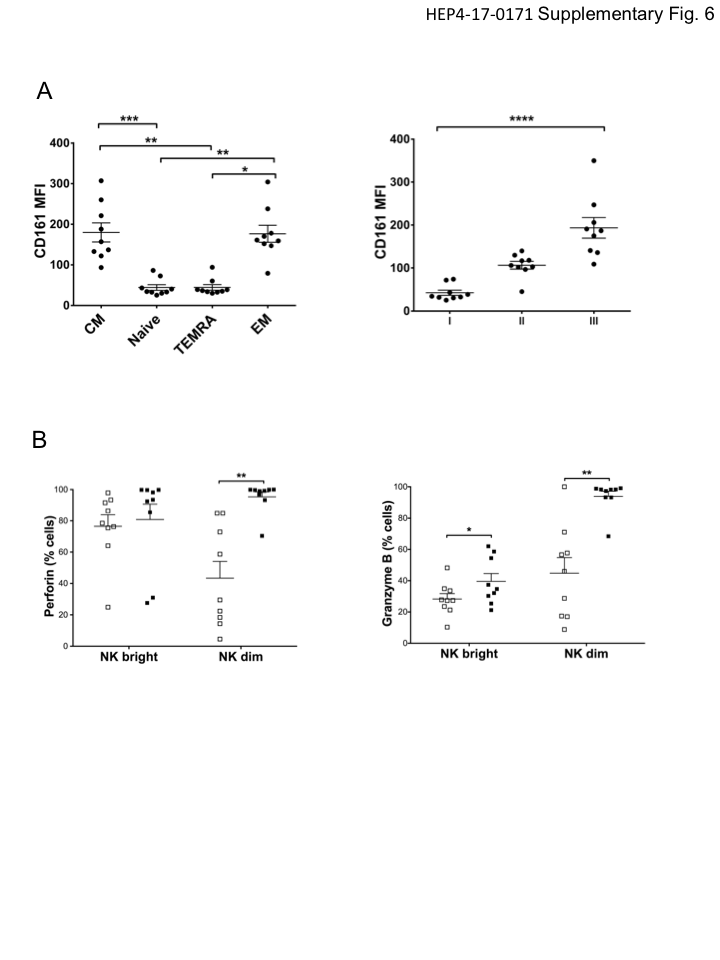

Supplement: Supplementary file 6 — Supporting Information Figure 6 [file HEP4-2-421-s006.tif]

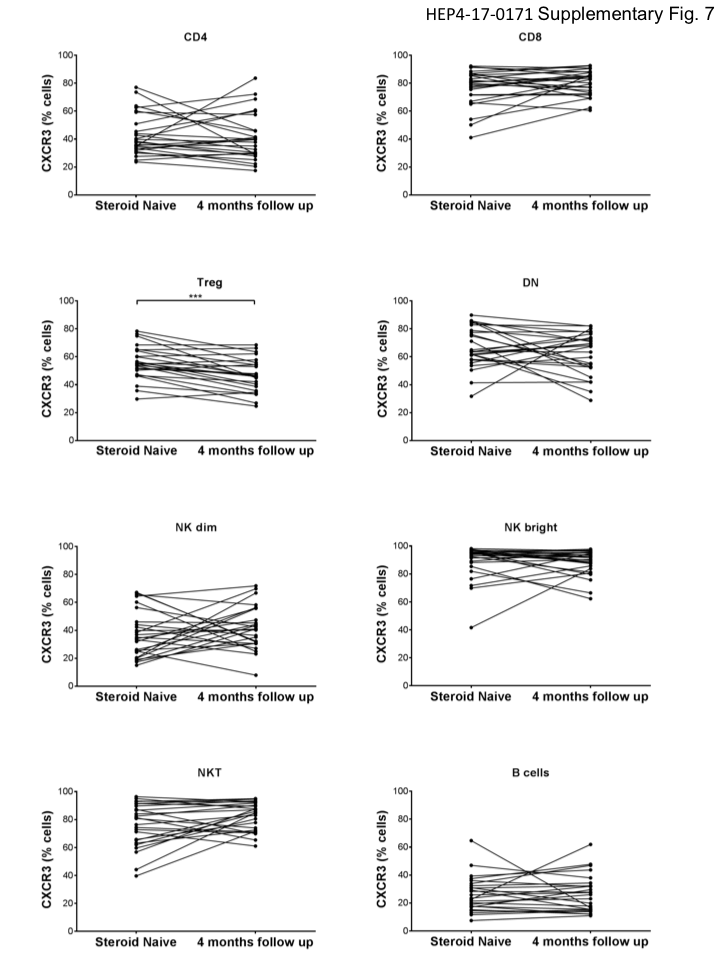

Supplement: Supplementary file 7 — Supporting Information Figure 7 [file HEP4-2-421-s007.tif]

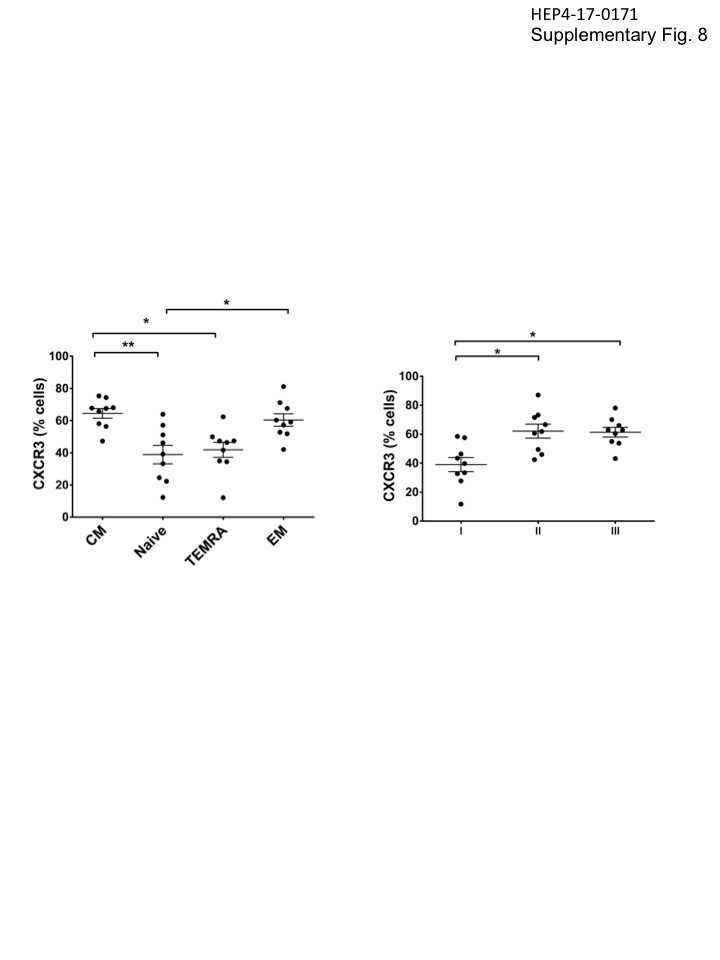

Supplement: Supplementary file 8 — Supporting Information Figure 8 [file HEP4-2-421-s008.tif]

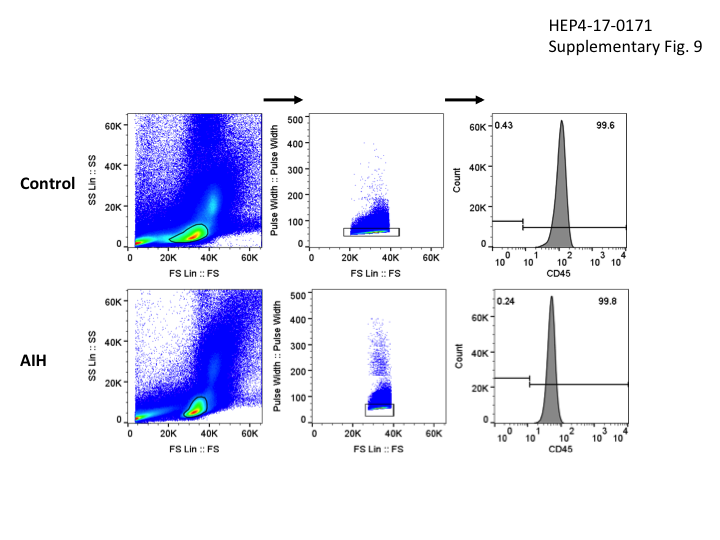

Supplement: Supplementary file 9 — Supporting Information Figure 9 [file HEP4-2-421-s009.tif]

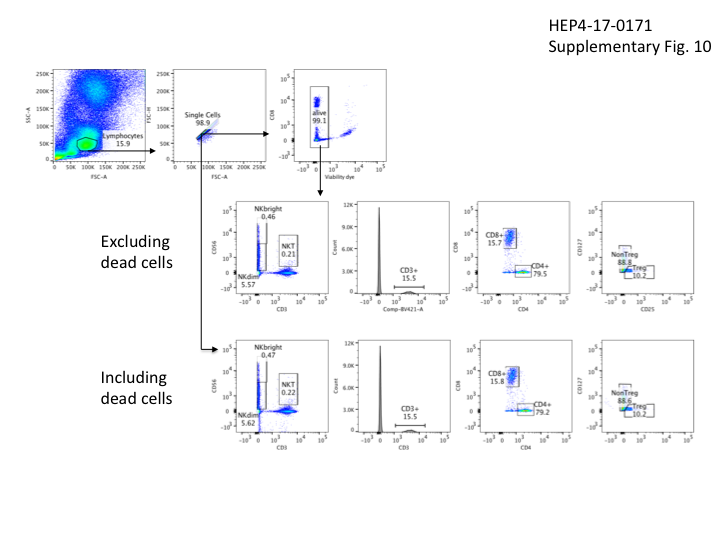

Supplement: Supplementary file 10 — Supporting Information Figure 10 [file HEP4-2-421-s010.tif]
