# Supplementary material for: Changes in natural killer cells and exhausted memory regulatory T Cells with corticosteroid therapy in acute autoimmune hepatitis
Source: Hepatol Commun. 2018 Feb 26;2(4):421–36. doi: 10.1002/hep4.1163 (PMC5880196; doi:10.1002/hep4.1163)
Supplement: Supplementary file 11 — Supporting Information Figures [file HEP4-2-421-s011.docx]

**Supplementary Figure 1:** Flow cytometry overlay of PD-1, IL-6R, CD161 and CXCR3 on immune subsets in the peripheral blood of AIH patients.

**Supplementary Figure 2: Frequencies of Treg and NKbright cells as proportions of the total lymphocyte population in the bloods of controls compared to patients with newly diagnosed AIH before and after 4 months immunosuppression treatment.** Steroid naïve and 4 months follow up frequencies were compared by Wilcoxon’s test (**** = *p* <0.0001). Frequencies in haemochromatosis controls were compared to each of steroid naïve and 4 months follow up by Mann-Whitney tests (** = *p*<0.01; *** = *p*<0.001). Error bars are median ± SEM.

**Supplementary Figure 3: Ratios of Treg to CD8, NK^dim^ and NKT cells in newly diagnosed AIH patients at baseline and after 4 months immunosuppression treatment.**

**Supplementary Figure 4: Treg phenotype in AIH before the start of corticosteroid therapy. (A)** Expression level (Median Fluorescence Intensity (MFI) of staining) of regulatory markers FOXP3, CTLA-4 and CD39 on memory and naïve subsets of Treg defined by CD45RA and CCR7: Central Memory (CM, CD45RA^neg^CCR7^+^), Effector Memory (EM, CD45RA^neg^CCR7^neg^), Naïve (CD45RA^+^CCR7^+^), Terminally differentiated tissue resident effector memory RA-positive (TEMRA, CD45RA^+^CCR7^-^). **(B)** Frequency and level (MFI) of regulatory marker expression by the Treg fractions defined by CD45RA and CD25: Fraction I (I, CD45RA^+^CD25^low^); Fraction II (II, CD45RA^neg^CD25^high^); Fraction III (III, CD45RA^neg^CD25^low^).

**Supplementary Figure 5:** Percentage CD161 expression by immune subsets in the peripheral blood of patients with newly diagnosed autoimmune hepatitis before steroid and 4 months after immunosuppression therapy.

**Supplementary Figure 6: (A) CD161 expression by Treg subsets in the peripheral blood of treatment naïve AIH patients.** Expression level (Median Fluorescence Intensity (MFI) of staining) of CD161 on CD161-expressing cells within each of the memory and naïve subsets and fractions of Treg defined by CD45RA and CCR7 or CD45RA and CD25 expression (Central Memory (CM, CD45RA^neg^CCR7^+^), Effector Memory (EM, CD45RA^neg^CCR7^neg^), Naïve (CD45RA^+^CCR7^+^), Terminally differentiated tissue resident effector memory RA-positive (TEMRA, CD45RA^+^CCR7^-^), Fraction I (I, CD45RA^+^CD25^low^), Fraction II (II, CD45RA^neg^CD25^high^), Fraction III (III, CD45RA^neg^CD25^low^)). **(B and C) Perforin and Granzyme B expression by CD161^pos^ and CD161^neg^ NK subsets.** Frequencies of expression of cytotoxic mediators granzyme B and perforin by NK cells within the CD161^pos^ and CD161^neg^ NK^bright^ and NK^dim^ subsets of CD3^neg^CD56^+^ NK cells in treatment naïve AIH.

**Supplementary Figure 7:** Percentage CXCR3 expression by immune subsets in the peripheral blood of patients with newly diagnosed AIH before steroid and 4 months after immunosuppression therapy.

**Supplementary Figure 8: Percentage** **CXCR3 expression by memory and naïve Treg subsets and Treg fractions in the peripheral blood of treatment naïve AIH patients.** Memory and naïve subsets were defined by CD45RA and CCR7: Central Memory (CM, CD45RA^neg^CCR7^+^), Effector Memory (EM, CD45RA^neg^CCR7^neg^), Naïve (CD45RA^+^CCR7^+^), Terminally differentiated tissue resident effector memory RA-positive (TEMRA, CD45RA^+^CCR7^-^). Fractions were defined by CD45RA and CD25: Fraction I (I, CD45RA^+^CD25^low^); Fraction II (II, CD45RA^neg^CD25^high^); Fraction III (III, CD45RA^neg^CD25^low^).

**Supplementary Figure 9:** Frequencies of CD45 expressing cells within the lymphocyte gate of peripheral blood mononuclear cell preparations isolated from control and Autoimmune Hepatitis patient bloods showing that cells within this gate are >99% CD45 expressing leukocytes. The position of the bidirectional gate indicates the division between cells stained positively for CD45 and unstained cells.

**Supplementary Figure 10:** Dead cell frequencies within the lymphocyte gate of peripheral blood mononuclear cells prepared according to the UK-AIH protocol were determined by the inclusion of viability dye staining prior to surface staining. The populations of immune subsets examined in the UK-AIH study were then stained and their frequencies determined when dead cells were either included or excluded in the gating strategy. One representative example is shown.
