# Supplementary material for: Changes in natural killer cells and exhausted memory regulatory T Cells with corticosteroid therapy in acute autoimmune hepatitis
Source: Hepatol Commun. 2018 Feb 26;2(4):421–36. doi: 10.1002/hep4.1163 (PMC5880196; doi:10.1002/hep4.1163)
Supplement: Supplementary file 12 — Supporting Information Tables [file HEP4-2-421-s012.docx]

**Supplementary Table 1:** Longitudinal clinical characteristics of the UK-AIH treatment naïve cohort followed up from baseline to four months

| \| **Patient** \| **Age** \| **Gender** \| **ALT (Units/L)** \| \| **Bilirubin (μmol/L)** \| \| **IgG (mg/dL)** \| \| **Steroid dose at baseline** \| **Steroid dose at 4 months** \| **Other immunosuppressant drugs given** \| \| --- \| --- \| --- \| --- \| --- \| --- \| --- \| --- \| --- \| --- \| --- \| --- \| \| Baseline \| 4 months \| Baseline \| 4 months \| Baseline \| 4 months \| \| 1 \| 56 \| Female \| 669 \| 128 \| 13 \| 8 \| 31.4 \| 15.4 \| Prednisolone (30mg) \| Prednisolone (20mg) \| AZA 25mg (month 4) \| \| 2 \| 53 \| Female \| 834 \| 30 \| 28 \| 8 \| 30.3 \| 14.3 \| Prednisolone (40mg) \| Prednisolone (8mg) \| AZA 100mg (month 4) \| \| 3 \| 67 \| Male \| 115 \| 25 \| 10 \| 7 \| 34.2 \| 14.4 \| Prednisolone (40mg) \| Prednisolone (5mg) \| AZA 75mg (month 4) \| \| 4 \| 73 \| Female \| 931 \| 21 \| 15 \| 9 \| 14 \| 11.8 \| Prednisolone (30mg) \| Prednisolone (25mg) \| AZA 50mg (week 4) \| \| 5 \| 44 \| Female \| 36 \| 9 \| 9 \| 7 \| 14.6 \| 8.3 \| Prednisolone (20mg) \| Prednisolone (5mg) \| AZA 100mg (week 2) \| \| 6 \| 72 \| Female \| 723 \| 17 \| 25 \| 23 \| 15.4 \| 10.2 \| Prednisolone (20mg) \| Prednisolone (7.5mg) \| UDCA \| \| 7 \| 38 \| Male \| 39 \| 24 \| 30 \| 30 \| 19.6 \| Not done \| Prednisolone (20mg) \| Prednisolone (10mg) \| None \| \| 8 \| 66 \| Female \| 21 \| 134 \| 6 \| 10 \| 13.8 \| 17.1 \| Prednisolone (20mg) \| Prednisolone (10mg) \| AZA (month 8) \| \| 9 \| 28 \| Female \| 104 \| 21 \| 10 \| 6 \| 20 \| 12.7 \| Prednisolone (30mg) \| Prednisolone (10mg) \| None \| \| 10 \| 68 \| Female \| 684 \| 43 \| 34 \| 13 \| 38.1 \| 16.1 \| Prednisolone (30mg) \| Prednisolone (5mg) \| AZA ( week 4 to month 4); MMF (month 8) \| \| 11 \| 44 \| Female \| 853 \| 11 \| 66 \| 7 \| 18.6 \| 12.1 \| Prednisolone (20mg) \| Prednisolone (7mg) \| AZA 100mg (week 2 to month 12) \| \| 12 \| 64 \| Male \| 96 \| 27 \| 18 \| 8 \| 33.3 \| 15 \| Prednisolone (20mg) \| Prednisolone (5mg) \| AZA 50mg (month 4) \| \| 13 \| 59 \| Female \| 173 \| 15 \| 65 \| 11 \| 35.6 \| 14.9 \| Prednisolone (20mg) \| Prednisolone (5mg) \| AZA 100mg (week 6) \| \| 14 \| 21 \| Female \| 173 \| 30 \| 47 \| 8 \| 17 \| 11 \| Prednisolone (20mg) \| Prednisolone (5mg) \| AZA 100mg (week 6) \| \| 15 \| 63 \| Female \| 69 \| 23 \| 12 \| 13 \| 10.5 \| Not done \| Prednisolone (30mg) \| Prednisolone 12.5mg \| AZA 50mg (month 4) \| \| 16 \| 56 \| Female \| 1148 \| 37 \| 118 \| ≤ 15 \| Neg \| Not done \| Prednisolone (30mg) \| Prednisolone (10mg) \| AZA 50mg (month 4) \| \| 17 \| 52 \| Female \| 747 \| 30 \| 17 \| 15 \| 25.6 \| Not done \| Prednisolone (20mg) \| Prednisolone (10mg) \| MMF 2g at month 4 \| \| 18 \| 71 \| Female \| 244 \| 35 \| 27 \| 12 \| 35.3 \| 12.8 \| Budesonide (9mg) \| Budesonide (9mg) \| 6MP 50mg (month 4) \| \| 19 \| 63 \| Female \| 231 \| 23 \| 25 \| 20 \| 12 \| Not done \| Budesonide (9mg) \| Not available \| AZA 125mg (month 4) \| \| 20 \| 68 \| Female \| 168 \| 17 \| 13 \| 12 \| 26 \| 11.2 \| Prednisolone (20mg) \| Prednisolone (12.5mg) \| AZA 75mg (month 4) \| \| 21 \| 46 \| Female \| 1352 \| 43 \| 28 \| 7 \| 20.3 \| 11.5 \| Budesonide (9mg) \| Budesonide (6mg) \| AZA 100mg (month 4) \| \| 22 \| 39 \| Female \| 113 \| 37 \| 14 \| 10 \| 24.6 \| 14 \| Budesonide (9mg) \| Budesonide (3mg) \| AZA 100mg (month 4) \| \| 23 \| 50 \| Female \| 101 \| 31 \| 11 \| 13 \| 17.7 \| 11.6 \| Prednisolone (30mg) \| Prednisolone (30mg) \| MMF 0.5g/day (month 4) \| \| 24 \| 58 \| Female \| 82 \| 30 \| 6 \| 6 \| 25.9 \| 11.9 \| Prednisolone (40mg) \| Prednisolone (30mg) \| AZA 25mg (month 4) \| \| 25 \| 54 \| Female \| 28 \| 37 \| 4 \| 6 \| 17.2 \| 12.9 \| Prednisolone (20mg) \| Prednisolone (15mg) \| AZA 50mg (month 4) \| \| 26 \| 45 \| Female \| 71 \| 21 \| 23 \| 18 \| Not done \| Not done \| Not available \| Not available \| Not available \| |
| --- | --- | --- | --- | --- | --- | --- | --- | --- | --- | --- | --- | --- | --- | --- | --- | --- | --- | --- | --- | --- | --- | --- | --- | --- | --- | --- | --- | --- | --- | --- | --- | --- | --- | --- | --- | --- | --- | --- | --- | --- | --- | --- | --- | --- | --- | --- | --- | --- | --- | --- | --- | --- | --- | --- | --- | --- | --- | --- | --- | --- | --- | --- | --- | --- | --- | --- | --- | --- | --- | --- | --- | --- | --- | --- | --- | --- | --- | --- | --- | --- | --- | --- | --- | --- | --- | --- | --- | --- | --- | --- | --- | --- | --- | --- | --- | --- | --- | --- | --- | --- | --- | --- | --- | --- | --- | --- | --- | --- | --- | --- | --- | --- | --- | --- | --- | --- | --- | --- | --- | --- | --- | --- | --- | --- | --- | --- | --- | --- | --- | --- | --- | --- | --- | --- | --- | --- | --- | --- | --- | --- | --- | --- | --- | --- | --- | --- | --- | --- | --- | --- | --- | --- | --- | --- | --- | --- | --- | --- | --- | --- | --- | --- | --- | --- | --- | --- | --- | --- | --- | --- | --- | --- | --- | --- | --- | --- | --- | --- | --- | --- | --- | --- | --- | --- | --- | --- | --- | --- | --- | --- | --- | --- | --- | --- | --- | --- | --- | --- | --- | --- | --- | --- | --- | --- | --- | --- | --- | --- | --- | --- | --- | --- | --- | --- | --- | --- | --- | --- | --- | --- | --- | --- | --- | --- | --- | --- | --- | --- | --- | --- | --- | --- | --- | --- | --- | --- | --- | --- | --- | --- | --- | --- | --- | --- | --- | --- | --- | --- | --- | --- | --- | --- | --- | --- | --- | --- | --- | --- | --- | --- | --- | --- | --- | --- | --- | --- | --- | --- | --- | --- | --- | --- | --- | --- | --- | --- | --- | --- | --- | --- | --- | --- | --- | --- | --- | --- | --- | --- | --- | --- | --- | --- | --- | --- | --- | --- | --- | --- | --- | --- | --- | --- | --- | --- | --- | --- | --- | --- | --- | --- | --- | --- | --- | --- | --- | --- | --- | --- | --- | --- | --- | --- | --- | --- | --- | --- | --- | --- | --- | --- |

**Supplementary Table 2:** Spearman’s correlation analyses for the changes in peripheral blood leukocyte subset frequencies in treatment naïve patient bloods between baseline and 4-months on steroid compared to the changes in ALT, Bilirubin and IgG during this period.

| \| **Change in Leukocyte Subset** \| **Change in ALT** \| \| \| **Change in Bilirubin** \| \| \| **Change in IgG** \| \| \| \| --- \| --- \| --- \| --- \| --- \| --- \| --- \| --- \| --- \| --- \| \| ***N*** \| ***Rho*** \| ***p-Value*** \| ***N*** \| ***Rho*** \| ***p-Value*** \| ***N*** \| ***Rho*** \| ***p-Value*** \| \| CD4 T cell \| 24 \| -0.008 \| 0.971 \| 23 \| 0.028 \| 0.898 \| 18 \| 0.061 \| 0.810 \| \| CD8 T cell \| 24 \| -0.121 \| 0.574 \| 23 \| -0.240 \| 0.270 \| 18 \| 0.189 \| 0.453 \| \| DN T cell \| 24 \| 0.097 \| 0.654 \| 23 \| -0.069 \| 0.753 \| 18 \| -0.201 \| 0.423 \| \| CD4+CD8+T cell \| 24 \| -0.187 \| 0.382 \| 23 \| -0.053 \| 0.812 \| 18 \| 0.020 \| 0.938 \| \| Treg \| 24 \| 0.228 \| 0.284 \| 23 \| -0.023 \| 0.918 \| 18 \| 0.179 \| 0.478 \| \| NK^dim^ \| 24 \| 0.214 \| 0.316 \| 23 \| 0.251 \| 0.248 \| 18 \| 0.290 \| 0.243 \| \| NK^bright^ \| 24 \| 0.462 \| 0.023 \| 23 \| 0.313 \| 0.146 \| 18 \| 0.121 \| 0.633 \| \| NKT cell \| 24 \| -0.060 \| 0.778 \| 23 \| -0.04 \| 0.857 \| 18 \| 0.232 \| 0.354 \| \| B cell \| 23 \| 0.282 \| 0.193 \| 22 \| -0.223 \| 0.319 \| 17 \| -0.316 \| 0.216 \| |
| --- | --- | --- | --- | --- | --- | --- | --- | --- | --- | --- | --- | --- | --- | --- | --- | --- | --- | --- | --- | --- | --- | --- | --- | --- | --- | --- | --- | --- | --- | --- | --- | --- | --- | --- | --- | --- | --- | --- | --- | --- | --- | --- | --- | --- | --- | --- | --- | --- | --- | --- | --- | --- | --- | --- | --- | --- | --- | --- | --- | --- | --- | --- | --- | --- | --- | --- | --- | --- | --- | --- | --- | --- | --- | --- | --- | --- | --- | --- | --- | --- | --- | --- | --- | --- | --- | --- | --- | --- | --- | --- | --- | --- | --- | --- | --- | --- | --- | --- | --- | --- | --- | --- | --- | --- | --- | --- | --- | --- | --- |

Shading indicates significant correlation

**Supplementary Table 3:** Frequencies of CD45-expressing cells within the lymphocyte gate of Peripheral blood mononuclear cells isolated from patients with Autoimmune Hepatitis and controls.

| Subject | Classification | CD45+ frequency (% cells) |
| --- | --- | --- |
| 1 | AIH | 99.8 |
| 2 | AIH | 99.2 |
| 3 | AIH | 99.9 |
| 4 | AIH | 98.8 |
| 5 | Control | 99.3 |
| 6 | Control | 99.4 |
| 7 | Control | 99.6 |

**Supplementary Table 4:**Frequencies of dead cells within the lymphocyte gate of peripheral blood mononuclear cells from two donors that had been prepared according to the UK-AIH protocol and the frequencies of the cell subsets analysed in the UK-AIH study when determined including or excluding dead cells in the gating strategy.

|  |  |  | Cell subset frequencies | | | | | | | |
| --- | --- | --- | --- | --- | --- | --- | --- | --- | --- | --- |
| Subject | Analysis | % singlet cells included | Nkbright (% total cells) | Nkdim (% total cells) | NKT (% total cells) | CD3+ (% total cells) | CD4+ (% CD3+ cells) | NonTreg (% CD4+ cells) | Treg (% CD4+ cells) | CD8+  (% CD3+ cells) |
| 1 | Gating including dead | 100.0 | 0.9 | 7.8 | 2.2 | 68.8 | 68.8 | 93.7 | 6.3 | 25.6 |
|  | Gating excluding dead | 97.3 | 0.8 | 7.4 | 1.9 | 69.8 | 68.8 | 93.7 | 6.3 | 25.7 |
|  | Difference between gatings (%) | 2.7 | 0.0 | 0.4 | 0.3 | -1.0 | 0.0 | 0.0 | 0.0 | -0.1 |
| 2 | Gating including dead | 100.0 | 0.5 | 5.6 | 0.2 | 15.5 | 79.2 | 88.6 | 10.2 | 15.8 |
|  | Gating excluding dead | 99.1 | 0.5 | 5.6 | 0.2 | 15.5 | 79.2 | 88.8 | 10.2 | 15.7 |
|  | Difference between gatings (%) | 0.9 | 0.0 | 0.0 | 0.0 | 0.0 | 0.0 | -0.2 | 0.0 | 0.1 |
