# Supplementary material for: Changes in natural killer cells and exhausted memory regulatory T Cells with corticosteroid therapy in acute autoimmune hepatitis
Source: Hepatol Commun. 2018 Feb 26;2(4):421–36. doi: 10.1002/hep4.1163 (PMC5880196; doi:10.1002/hep4.1163)
Supplement: Supplementary file 13 — Supporting Information [file HEP4-2-421-s013.docx]

**Isolation of peripheral blood mononuclear cells (PBMC) and multi-colour flow cytometry to define the peripheral immune phenotype of AIH patients.**

Peripheral blood mononuclear cells were isolated from the whole blood by density gradient separation using Lympholyte (VH Bio Ltd) at 800 x *g* for 20 minutes. The mononuclear layer was collected and washed twice with Phosphate Buffered Saline.

3.5 - 7.5×10^5^ cells were used per stain. Cells were aliquotted to 5ml polypropylene round bottom tubes (Falcon) tubes and antibodies to surface antigens added in 2% foetal calf serum (FCS) (Sigma Aldrich) in PBS buffer. After 30 minutes incubation on ice, excess antibodies were removed by a 3ml wash in 2%-FCS buffer and cells phenotyped for surface marker expression only were fixed by 10-minute incubation at room temperature in 3% formadehyde solution. Fixative was removed by washing in PBS and cells resuspended in PBS for analysis within 24-hours. Cells analysed for expression of intracellular proteins were fixed and stained with fluorophore conjugated antibodies to the intracellular markers using the FOXP3/Transcription factor staining set (eBioscience) according to manufacturer’s instructions. Data were acquired using a CyAN ADP flow cytometer. Single-fluorophore-labelled anti-mouse IgGκ/negative control (FBS) compensation particles (BD Biosciences) were used for compensation. Data were analysed offline using FlowJo (Tree Star Inc., Ashland, OR). Expression frequencies (percentage of cells presenting with staining greater than that of an isotype matched control) and/or median fluorescence intensity of staining (MFI) values, which provide measurement of the level of marker abundance per expressing cell, are reported.

Using a panel of antibodies to the surface receptors CD3, CD4, CD8, CD56, CD19, CD25 and CD127 we were able to define cell subsets including CD4, CD8, CD4^neg^CD8^neg^ (double-negative (DN)) T cells, CD4^pos^CD25^pos^CD127^neg^ Treg, CD19^pos^ B cells, CD3^pos^CD56^pos^ Natural Killer T cells (“NKT”) and CD3^neg^CD56^bright^ (NK^bright^), and CD3^neg^CD56^dim^ (NK^dim^) cells (Figure 1A) and identify the expression of cell functional surface markers including CXCR3, IL-6R, CD161, and PD1 selected for roles in lymphocyte liver homing, plasticity and exhaustion (Supplementary Figure 1). The anti-human antibodies used in flow cytometric analysis of lymphocyte subsets and marker expressions included anti-CD3-PeCy7 (SK7, BD Biosciences), anti-CD3-Vioblue and anti-CD3-Viogreen (both BW264/56, Miltenyi Biotec), anti-CD4-PerCP/Cy5.5 (RPA-T4, eBioscience), anti-CD4-Viogreen (VIT4, Miltenyi Biotec), anti-CD8 PerCPCy5.5Vio700 (BW135/80, Miltenyi Biotec), anti-CD19 APCVio770 (LT19, Miltenyi Biotec), anti-CD25-BV421 (M-A251, BD Biosciences), anti-CD25-APC (4E3, Miltenyi Biotec), anti-CD56-PeVio770 (REA196, Miltenyi Biotec), anti-CD45Ra-APCVio770 (T6D11, Miltenyi Biotec), anti-CD127-FITC (MB15-18C9), anti-CXCR3-PE (G025H7, Biolegend), anti-IL-6R-PE (M5, BD Biosciences), anti-CD161-PE and anti-CD161-APC (both 191B8, Miltenyi Biotec), anti-PD1-PE (eBioJ105, eBiosciences), anti-CCR7-PE-CF594 (150503, BD Biosciences), anti-Granzyme B-FITC (REA226, Miltenyi-biotec), anti-Perforin-PE (dG9, eBioscience), anti-CTLA-4-PE (BN13, BD Biosciences), anti-FOXP3-APC (PCH101, eBioscience) and anti-CD39-PE (A1, eBioscience).

**Flow cytometry phenotyping of liver infiltrating immune cells for expression of Lectin-like transcript-1.**

After staining dead cells with the e506 viability dye (eBioscience, Fisher Scientific) and blocking Fc receptors by incubation with human TruStain FcX^TM^ (Biolegend) in 2% FCS buffer according to manufacturer’s instructions, fluorochrome conjugated antibodies to anti-LLT1 PE (R402659, R and D Systems) or isotype control as well as subset defining markers (anti-CD3 APC (UCHT1, BD Biosciences), anti-CD19 APCVio770 (LT19, Miltenyi Biotec), anti-CD14 Vioblue (TÜK4, Miltenyi Biotec), anti-CD16 FITC (REA423, Miltenyi-Biotec) and anti-CD66b PeCy7 (G10F5, Biolegend) were added and cells stained on ice for 30-minutes before fixation in 3% formaldehyde and data acquisition on a CyAN ADP flow cytometer.

**NK and T cell co-culture assays**

CD3^neg^CD56^pos^ NK cells were sorted from PBMC using the NK cell enrichment kit (StemCell) according to manufacturer’s instructions. CD4^pos^CD25^pos^CD127^neg^ Treg and CD4^pos^CD25^neg^CD127^pos^ non-Treg were isolated into separate populations from PBMC of the same donor by CD4 enrichment using the Mojo CD4 enrichment kit (BioLegend) followed by fluorochrome labelling with anti-CD3 Viogreen (BW264/56, Miltenyi Biotec), anti-CD127-FITC (MB15-18C9, Miltenyi Biotec), anti-CD25 PE (4E3, Miltenyi Biotec), and anti-CD4-PerCP/Cy5.5 (RPA-T4, eBioscience), and flow sorting on a BD FACSAria. The amount of blood volume required to freshly isolate both NK and Treg is more than 200ml for this functional assay thus we could use haemochromatosis patient bloods only for this study. After isolation, cells were suspended in RPMI medium supplemented with 10% Heat inactivated FCS (Sigma), 2mM Glutamine, 100IU/ml Penicillin and 100IU/ml Streptomycin (GIBCO, invitrogen). Cultures of NK alone, NK + Treg (1:1) and NK + Non-Treg (1:1) were set up without treatment and in the presence of IL-12 (20ng/ml) + IL-15 (20ng/ml) (Peprotech). Treg suppression inspector (CD3/CD28/CD2) beads (Miltenyi biotec) were added in proportion to the number of T cells to activate the T cells. Following overnight (co)culture anti-CD107a PE (H4A3, BD biosciences) or Isotype control antibodies were added to the cultures to mark cells undergoing degranulation and after 1hour Brefeldin A (5μg/ml) was added for 5 hours to block cytokine release. Dead cells in the culture were then dyed with NIH zombie reagent (BioLegend), and after washing, surface CD3 and CD56 were labelled using anti-CD3 Viogreen (BW264/56, Miltenyi Biotec) and anti-CD56-PeVio770 (REA196, Miltenyi Biotec). Following 10-minute fixation in 3% formaldehyde, cells were permeabilised with 0.1% saponin solution and stained intracellularly with antibodies anti-CD4-PerCPCy5.5 (RPA-T4, Biolegend) anti-TNFα-FITC (MAb11, eBiosciences/Fisher Scientific), and anti-IFNy-APC (B27, BD Biosciences). Cells were acquired on a CyAN ADP flow cytometer and data analysed using Flowjo version-10 software. Frequencies of IFNy, TNFα and CD107a expression by CD3^neg^CD4^neg^CD56^pos^ NK cells were quantified for each condition.
